# H syndrome: 5 new cases from the United States with novel features and responses to therapy

**DOI:** 10.1186/s12969-017-0204-y

**Published:** 2017-10-17

**Authors:** Jessica L. Bloom, Clara Lin, Lisa Imundo, Stephen Guthery, Shelly Stepenaskie, Csaba Galambos, Amy Lowichik, John F. Bohnsack

**Affiliations:** 1 Department of Pediatrics, University of Colorado, Aurora, CO 80045 USA; 2 Department of Pediatrics, Columbia University College of Physicians and Surgeons, New York, NY 10032 USA; 3 Department of Pediatrics, University of Utah, Salt Lake City, UT 84113 USA; 4 Department of Pathology and Dermatology, University of New Mexico, Albuquerque, NM 87102 USA; 5 Department of Pathology, University of Colorado, Aurora, CO 80045 USA; 6 Department of Pathology, University of Utah, Salt Lake City, UT 84113 USA

**Keywords:** H syndrome, SLC29A3, Autoinflammatory, Arthritis, Hyperpigmentation, Pediatric rheumatology, Genetic disorder, Biologic agents

## Abstract

**Background:**

H Syndrome is an autosomal recessive disorder characterized by cutaneous hyperpigmentation, hypertrichosis, and induration with numerous systemic manifestations. The syndrome is caused by mutations in *SLC29A3*, a gene located on chromosome 10q23, which encodes the human equilibrative transporter 3 (hENT3). Less than 100 patients with H syndrome have been described in the literature, with the majority being of Arab descent, and only a few from North America.

**Case presentation:**

Here we report five pediatric patients from three medical centers in the United States who were identified to have H syndrome by whole exome sequencing. These five patients, all of whom presented to pediatric rheumatologists prior to diagnosis, include two of Northern European descent, bringing the total number of Caucasian patients described to three. The patients share many of the characteristics previously reported with H syndrome, including hyperpigmentation, hypertrichosis, short stature, insulin-dependent diabetes, arthritis and systemic inflammation, as well as some novel features, including selective IgG subclass deficiency and autoimmune hepatitis. They share genetic mutations previously described in patients of the same ethnic background, as well as a novel mutation. In two patients, treatment with prednisone improved inflammation, however both patients flared once prednisone was tapered. In one of these patients, treatment with tocilizumab alone resulted in marked improvement in systemic inflammation and growth. The other had partial response to prednisone, azathioprine, and TNF inhibition; thus, his anti-TNF biologic was recently switched to tocilizumab due to persistent polyarthritis. Another patient improved on Methotrexate, with further improvement after the addition of tocilizumab.

**Conclusion:**

H syndrome is a rare autoinflammatory syndrome with pleiotropic manifestations that affect multiple organ systems and is often mistaken for other conditions. Rheumatologists should be aware of this syndrome and its association with arthritis. It should be considered in patients with short stature and systemic inflammation, particularly with cutaneous findings. Some patients respond to treatment with biologics alone or in combination with other immune suppressants; in particular, treatment of systemic inflammation with IL-6 blockade appears to be promising. Overall, better identification and understanding of the pathophysiology may help devise earlier diagnosis and better treatment strategies.

## Background

H Syndrome (OMIM #612391) is an autosomal recessive disorder characterized by cutaneous hyperpigmentation, hypertrichosis, and induration with numerous systemic manifestations [1, 2]. Coined in 2008 by Molho-Pessach et al., the syndrome is named for its most common clinical features: hyperpigmentation, hypertrichosis, hepatosplenomegaly, hearing loss, heart anomalies, hypogonadism, low height, hyperglycemia (insulin-dependent diabetes mellitus), and hallux valgus/flexion contractures [1]. While not always present, the pathognomonic skin findings most often involve the lower limbs, especially the inner thighs, but can appear throughout the body. The histologic correlates to these dermatologic findings include epidermal hyperplasia and increased basal pigmentation, and a dermal infiltrate including histiocytes, lymphocytes, and plasma cells, sometimes accompanied by hemosiderin deposition or calcification. Rosai-Dorfman disease (RDD) and related conditions predisposing to RDD or RDD-like lesions, including H syndrome, are classified as histiocytoses of the R group per a recent revised classification of histiocytoses and related neoplasms by the Histiocyte Society [3].

The most common features apart from skin findings include flexion contractures of fingers and toes, sensorineural hearing loss, short stature, and hepatosplenomegaly respectively followed by insulin-dependent diabetes mellitus (IDDM), lymphadenopathy, and microcytic anemia. Hypogonadism, azoospermia, and micropenis can also be seen as well as chronic diarrhea, which is often secondary to pancreatic dysfunction. There have been reports of patients with recurrent fever, some of which are accompanied by joint inflammation [4–7]. Laboratory results often reveal chronic elevation of inflammatory markers [2].

The syndrome is caused by homozygous or compound heterozygous mutations in *SLC29A3*, a gene on chromosome 10q22 that encodes the human equilibrative nucleoside transporter 3 (hENT3). This transporter helps with passive sodium-independent transportation of nucleosides and is critical for nucleotide synthesis by salvage pathways [8]. Multiple missense, nonsense, compound and deletion mutations of *SLC29A3* can lead to H syndrome, partly accounting for its large inter-familial variability. Thus, multiple phenotypically-varying disorders previously thought to be separate entities are now considered one condition; these include pigmented hypertrichosis with IDDM syndrome, familial histiocytosis syndromes (including Faisalbad histiocytosis), dysosteosclerosis, POEMS, and familial rhinosclerema [2, 9, 10].

To our knowledge, there have been about 90 patients confirmed to have H Syndrome worldwide. Most patients described in the literature have been of Arab descent, with only one noted to be Caucasian. Only a few patients with *SLC29A3* mutations have been identified at North American centers. In this report, we present five pediatric patients recently diagnosed with H Syndrome in three medical centers in the United States. One patient has autoimmune hepatitis with positive serologies, a finding not previously reported in H Syndrome. This patient, along with another, also suffers from polyarthritis; notably, only two cases of arthritis have been reported independent of fever [11, 12]. These cases illustrate the auto-inflammatory nature of H syndrome while all 5 cases add further information about its clinical phenotype and response to treatment.

## Case presentations

### Clinical findings

#### Patient 1

This male was born in New Mexico to a 23 year old G1P0 female at 40 weeks gestation via normal spontaneous vaginal delivery after an uncomplicated pregnancy. He was small for gestational age, weighing 2620 g (~3rd percentile) with APGARS of 8 and 9. Both parents were of Hispanic descent with no family history of birth defects, consanguinity, stillbirths, or developmental delay, apart from a half-paternal aunt with Rett Syndrome.

**Fig. 1:**
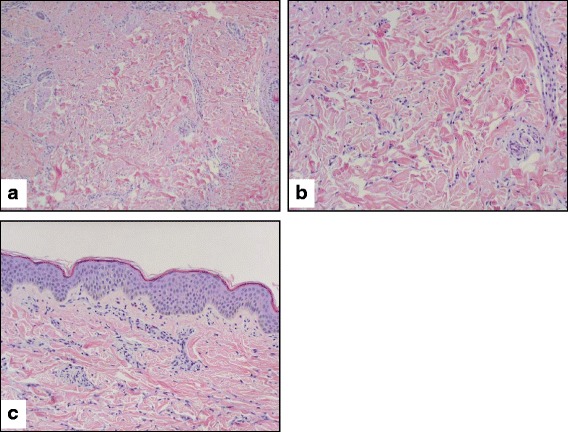
Patient 1 Skin Biopsy. **a**: The dermis contains scattered interstitial histiocytes and a few background eosinophils and mast cells. (10X) **b:** Higher magnification showing a histiocytic infiltration in the dermis. These histiocytes stain positive for CD68, factor XIIIa, Ham56, and S100P (subset) but negative for CD1a. (20X) **c:** The overlying epidermis is not involved. No hyperpigmentation is appreciated. (20X)

At 6.5 months of age, he developed non-bilious non-bloody emesis and diarrhea, followed within a week by jaundice, decreased appetite and low-grade fevers to 101 F. On exam, his height and weight were less than the 3rd percentile with a head circumference at the 42nd percentile. He had mild jaundice, frontal bossing, prominent scalp veins, an enlarged anterior fontanelle, hepatomegaly, and multiple bluish non-blanching lesions on his trunk that became erythematous over a week’s time. Significant laboratory findings included elevated transaminases (five times the upper limit of normal), lipase 381 U/L (normal 0–290), CRP 70 mg/L (0–10), microcytic anemia, mild iron deficiency, neutropenia and thrombocytosis with normal bilirubin, alkaline phosphatase, prothrombin and partial thromboplastin times. Stool culture was negative for salmonella, shigella, and campylobacter while serum antibodies to EBV, Parvovirus, and Hepatitis A, B, and C were negative. CMV IgG antibody was positive, while the serum CMV IgM antibody was negative. Ultrasound showed multiple hypoechoic lesions in the spleen in an infiltrative pattern. A brain MRI demonstrated mild prominence of his subarachnoid space without significant ventriculomegaly. Biopsies of his skin nodules revealed interstitial granulomatous dermatitis (+CD68, CD163, CD45, and CD4; − CD1a, CD117, S100, CD34, lysozyme, and MPO) without fungal or mycobacterial organisms and no involvement of the overlying epidermis (Fig. 1).

At 10 months of age, he had a generalized eczematous eruption on his trunk, extremities, and face along with a residual light brown patchy dyspigmentation on his trunk. Repeat skin biopsies demonstrated similar dermal involvement as his previous biopsy (interstitial histiocytic infiltrate with scattered eosinophils) with new epidermal involvement described as eosinophilic spongiosis with vesicles, few neutrophils, and overlying parakeratosis.

At 17 months old, he was hospitalized for worsening failure to thrive (weight < 2nd percentile, height < 0.5th percentile, head circumference > 98th percentile), and regression from walking. Dysmorphic features were noted including frontal bossing, macrocephaly, mid-face hypoplasia, a low nasal bridge, pectus carinatum, rib flaring, rhizomelia, brachydactyly, hirsutism of his face and back, and hyperpigmentation of his back. Arthritis of his wrists, knees, and ankles as well as diffuse swelling of his fingers and dorsal hands were noted. An ophthalmologic exam showed no evidence of uveitis. He had normal social and language development. Abnormal laboratory findings included ESR 101 mm/h (<15), CRP 7.7 mg/dL (<1), AST 146 (20–60), ALT 123 (5–45), albumin 3.1 (3.4–4.2), IgA 284 (9–137), hemoglobin 10.1, and MCV 67.7. He had a normal bilirubin, GGT, white blood cell count, and platelet count. ANA was positive at 1:160 with negative specificities (Smith/RNP, dsDNA, SSA/SSB, centromere), anti-actin IgG was elevated at 29.3 (normal <19.9), and anti-LKM antibodies were negative. Evaluation for other etiologies, including RPR, HIV, CMV, Hepatitis B, Hepatitis C, PPD, TSH, T4, and alpha-1 antitrypsin profile were unrevealing. X-rays of his wrists, hands, knees, ankles and feet were unremarkable apart from demineralization. A skeletal survey revealed prominent growth arrest lines with mild thickening of the ribs and a J-shaped sella with a normal sized sella turcica. Abdominal ultrasound demonstrated resolution of the hypoechoic splenic parenchyma seen on previous ultrasound with no signs of hepatosplenomegaly.

**Fig. 2:**
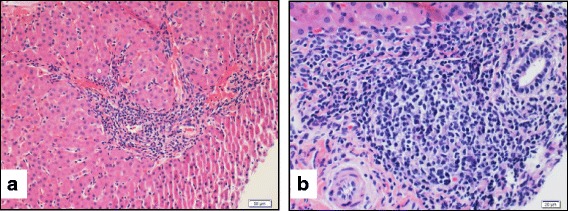
Patient 1 Liver Biopsy. **a**: Medium magnification of a hematoxylin-eosin stained core of the liver biopsy shows mildly expanded portal tract by a mild to moderate amount of inflammatory infiltrate. The inflammation, in places, extends to the interface. **b:** High magnification image shows that the inflammatory infiltrate is composed of a predominantly lymphocytic infiltrate. Rare eosinophils and plasma cells are noted

A liver biopsy revealed mild to moderate chronic active hepatitis (primarily lymphocytic without plasma cells) and interface hepatitis without significant fibrosis, viral inclusions, or ultrastructural abnormalities (Fig. 2).

At age 2, audiology studies detected mild right-sided hearing loss.

#### Patient 2

The proband is a 19-year-old male of Northern European extraction, the first child of non-consanguineous parents. Born full term at 3550 g, he was noted to have a two-vessel cord at birth and a murmur due to a bicuspid aortic valve. CT scan showed an absent inferior vena cava and a continuous dominant hemiazygos vein that drained into the azygos vein at the level of the T9 vertebral body. The azygos vein drained the right kidney. At his most recent cardiac evaluation at 18 years of age, he had stable dilation of his aorta without significant aortic stenosis or insufficiency. He has also had two episodes of symptomatic pericarditis.

A small area of hypertrichosis and hyperpigmentation in the lumbar area of his back was noted as an infant. This patch increased in size and extended to his anterior thighs and shins as he grew older.

He was noted to have localized swelling in his scrotum during his first year of life. Over time, the scrotum and mons pubis became indurated and tense. Ultrasound at age 17 months revealed normal appearing testes with echogenic material filling the scrotum and lining the wall, particularly the right epididymis. The findings were felt to be consistent with calcified meconium from meconium peritonitis. The scrotal mass increased in size until the whole scrotum was noticeably distended and by 4 years of age, the swelling also involved the suprapubic area. CT of his pelvis showed diffuse increased attenuation involving the subcutaneous fat from the level of the iliac crests to the upper thighs. An MRI at age 6 revealed bilateral inguinal lymphadenopathy.

**Fig. 3:**
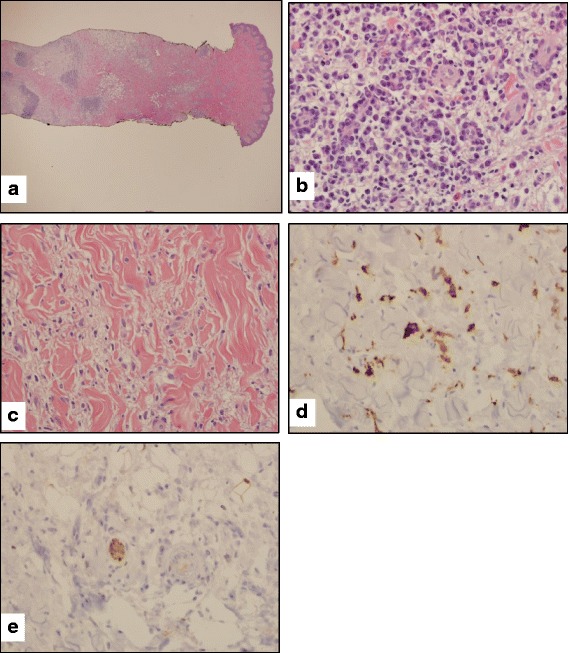
Patient 2, skin punch biopsy. **a.** Low-power view of skin overlying mons pubis shows patchy edema and chronic inflammation of the deep dermis and subcutaneous tissue with focal lymphoplasmacytic aggregates as well as mononuclear inflammatory cells dispersed within the interstitium. **b.** High-power view of deep dermal inflammatory aggregates shows prominent perivascular lymphoplasmacytic cuffing as has been described in cutaneous Rosai-Dorfman and related diseases. **c.** Foci of dermal edema contain scattered mononuclear cells including histiocytes, although emperipolesis is not appreciated. **d.** Immunohistochemistry for CD163 confirms the presence of dermal histiocytes, with occasional larger histiocytes highlighted. **e**. The histiocytes noted in 3D are not highlighted by immunohistochemistry for S100 protein; a peripheral nerve in the center of the field serves as a positive internal control

A deep skin punch biopsy from patient 2 showed mild epidermal hyperplasia with relatively mild basal hyperpigmentation and a striking infiltrate in the deep dermis and subcutis accompanied by edema and rare hemosiderin deposition. Cellular aggregates seen at low power (Fig. 3a) included a predominance of plasma cells with prominent perivascular cuffing (Fig. 3b), in addition to other mononuclear cells. Immunohistochemical stains for kappa and lambda showed a polyclonal phenotype, although with inversion of the expected kappa:lambda ratio (not shown). The plasma cells did not show strong immunoreactivity for IgG4 (not shown). Immunohistochemical staining for histiocytic markers showed diffuse staining of cells for CD68 and CD163, which were often present between the plasmacytic aggregates (Fig. 3c, d). However, these histiocytes were not markedly enlarged, did not show significant emperipolesis, and were not highlighted by immunohistochemical staining for S-100 protein (Fig. 3e).

The patient has always been short for his age. At 4 months old, his height was at the 10-20th percentile, which fell to <5th percentile by 9 months and to <3rd percentile by 5 years, at which time he was diagnosed with growth hormone deficiency. He had mildly delayed bone age throughout childhood and adolescence with no pituitary mass seen on imaging. A DEXA scan at 9 years of age showed normal bone mineral density.

He had a normal hearing screening at birth but developed significant hearing loss by 14 months of age and eventually had bilateral cochlear implants placed. He had enlargement of the lateral and third ventricles as well as the bifrontal and bitemporal extra-axial fluid spaces on brain MRI at 18 months of age consistent with cerebral volume loss. Arcus senilis was noted at 4 years of age. Two years later, he was described by a dysmorphologist to have a prominent forehead with a normal nasal root (OFC 50th percentile), mild hypertelorism and ptosis, prominence to his eyes, and a cowlick with a whorl on the right side of his forehead. External ears were slightly posteriorly rotated, but otherwise normal. At age 7, his hearing worsened and by a year later, he had the first of his now bilateral cochlear implants.

He had episodes of abdominal pain and loose stools throughout childhood. Upper endoscopy and colonoscopy performed at age 11 showed partial lactase deficiency in his duodenal mucosa and lymphoid hyperplasia throughout the colon with a normal terminal ileum biopsy. CT enteroclysis of the abdomen and pelvis at age 15 was unremarkable.

At age 15, he developed pain and stiffness in multiple joints, especially ankles and knees. He had proximal interphalangeal joint contractures in his 5th digits bilaterally and loss of range of motion in both ankles and knees. Left hand films showed normal mineralization and no erosions or joint space narrowing.

#### Patient 3

Patient 3 is the sister of Patient 2. She was born full-term with a birth weight of 3200 g. She was diagnosed with IDDM at age 4, and with celiac disease at age 8 based on antibody testing alone. While initially at the 50th percentile for height at 4 months of age, she fell to the 6th percentile by 5 years and less than the 1st percentile by 8. Head circumference remained at the 50th percentile during the first 3 years of life. She was diagnosed with H Syndrome by whole exome sequencing at 8 years of age. Hearing loss has been documented by hearing screens and she now wears hearing aids. She developed flexion contractures of her fifth PIPs between the ages of 9 and 10 years. She does not have dysmorphic features or cutaneous changes typical of H syndrome.

#### Patient 4

Patient 4 is a Caucasian, Sephardic Jew initially diagnosed with IDDM at 3 years of age. At age 12, she was seen by a pediatric rheumatologist for acute arthritis and diagnosed with post-streptococcal reactive arthritis. Her arthritis resolved after a year. At age 13, she developed a bruise-like morphea rash on her trunk, back and extremities. The rash consisted of hyperpigmented lesions, some waxy in nature, and hypertrichosis. Skin biopsies at age 14 revealed a panniculitis-like lymphoplasmacytic infiitrate localized to the subcutis. She received care from a dermatologist from age 16 to 19 for presumed morphea. At age 19, skin biopsies showed mild chronic inflammation of the dermis, not consistent with morphea nor were histiocytes present. Whole exome sequencing at age 20 years resulted in a genetic diagnosis of H Syndrome.

#### Patient 5

Patient 5, sibling to patient 4, was diagnosed with IDDM before the age of 5. At age 5, she began to have skin infiltrates and adenopathy. She was initially seen at a cancer center and underwent removal of several skin lesions over 3 years that were found to have normal, benign pathology; thus, no treatment was initiated. At age 10, she was noted to have progressive joint contractures and short stature. She also discovered 2–3 mildly hyperpigmented lesions on her back and thigh 2–4 cm in size with scant but long, coarse hairs. At age 14, a 2 cm mass appeared to increase in size on the bridge of her nose extending into the sinuses. Biopsy revealed respiratory mucosa with florid inflammatory infiltrate without evidence of neoplasm or a histiocytic process. The lesion could not be removed surgically.

Whole exome sequencing at 15 years old led to a diagnosis of H Syndrome. She was seen by a pediatric rheumatologist for active synovitis and contractures of her fingers. MRI with contrast revealed mild hyperemia of the distal growth plate of the fifth metacarpal bone, fifth metacarpophalangeal joint, and fifth PIP joint consistent with inflammatory arthritis but no definite synovitis. Mild T2 hyperintensity without accompanying enhancement of the distal growth plate was observed in the second metacarpal. No synovial enhancement was seen following the administration of gadolinium.

Summary of the clinical findings and laboratory results are found in Tables 1 and 2.

**Table 1:** Clinical features

Characteristic			Family 1	Family 2		Family 3	
			Patient 1	Patient 2	Patient 3	Patient 4	Patient 5
Origin			Hispanic	Caucasian	Caucasian	Caucasian, Sephardic Jew	Caucasian, Sephardic Jew
Gender			Male	Male	Female	Female	Female
Age of first manifestation			6 months	6 months	4 years 2 months	12 years	5 years
Age at diagnosis			2 years 5 months	18 years	8 years 4 months	20 years	15 years
Current Age			3 years	19 years	9 years	20 years	16 years
Manifestations	Incidence						
	Literature^a^	This Series					
Cutaneous Hyperpigmentation/Hypertrichosis	68%	80%	+	+	–	+	+
Flexion Contractures of Fingers or Toes	56%	80%	+	+	+	–	+
Hearing loss	53%	60%	+	+	+	–	–
Short Stature	49%	80%	+	+	+	–	+
Exophthalmos/ Proptosis/ Eyelid Swelling	28%	20%	–	+	–	+/−	–
Insulin-Dependent Diabetes Mellitus	23%	60%	–	–	+	+	+
Flat foot/ Foot Deformity	20%	20%	–	+	–	–	–
Arthritis	8%	80%	+	+	–	+	+
Hydrocephalus/ Benign Intracranial Hypertension/ Brain Edema	5%	40%	+	+	–	–	–
Macrocephaly/ Frontal Bossing	(not given)	40%	+	+	–	–	–
Additional Findings (with Incidence from the Literature)^a^	Patient 1: Renal anomaly (6%), Hepatomegaly (43%), Autoimmune hepatitis		Patient 2: Scrotal mass, Cardiac anomalies (34%), IgG subclass deficiency, Hypertriglyceridemia (4%), Recurrent fever (5%), Gluteal lipodystrophy (6%), Arcus Senilus (14%), Gastrointestinal involvement (15%), Lymphadenopathy (24%), Absent IVC, Recurrent pericarditis				Patient 5: Respiratory and nasal mucosa swelling (10%)

^a^as reported in Molho-Pessach, V. et al. (2014)

**Table 2:** Laboratory Findings

Laboratory Test	Patient 1	Patient 2	Patient 3	Patient 4	Patient 5
SLC29A3 mutation	c.1087C > T	c.347 T > G c.610 + 1G > C	c.347 T > G c.610 + 1G > C	G437R IVS2 + IG > C	G437R IVS2 + IG > C
Autoantibodies					
ANA	+ (1:640)	–		–	–
ANA specificities	–	- SCl 70		–	–
Celiac Disease	–	–	172 (0–19)^a^	–	–
Type I Diabetes Mellitus			+^b^	+^c^	+^c^
Thyroid	–		–	–	–
Anti-Actin	+29.3 (<19.9)			+21 (<19)	–
HSP70 (68 kDa)			*weak* +		
Anti-Cyclic Citrullinated Peptide	–	–		–	–
Rheumatoid Factor	–	–		–	–
Hematologic					
Microcytic Anemia	+	+	–	–	–
Elevated platelets	+	+	+	–	–
Elevated transaminases	+	–	–	–	–
Elevated ESR	+	+	+	+	+
Elevated CRP	+	+	+	+	+
Lipids					
HDL		4 mg/dL		25 mg/dL	24 mg/dL
Immunologic					
IgG	1182 mg/dL (413–1112)	2190 mg/dL (608–1229)	1077 mg/dL (584–1509)	1820 mg/dL (549–1584)	Nml
IgA	284 mg/dL (9–137)	32 mg/dL (68–378)	311 mg/dL (45–234)	Nml	342 mg/dL (42–249)
IgM	169 mg/dL (30–146)	66 mg/dL (48–226)	60 mg/dL (49–230)	Nml	Nml
IgG subclasses		IgG2, IgG4 undetectable	Nml	IgG-1 1430 (240–1118}	Nml
IgD		<0.2			
Endocrine					
GH		0.31 ng/ml (4.8 with hypoglycemia)	3.03 ng/ml		
IGF BP3	1.5 mcg/mL (0.7–3.6)	1.18 mg/ml (1.16–3.13)	2490 ng/ml (2314–6086)		
IGF-1	146 ng/mL (51–303)	27 ng/ml (109–485)	58 ng/ml (64–358)		
TSH, free T4	Nml	Nml	Nml	Nml	Nml

Key: (−) = Negative, (+) = Positive, Blank = Not Tested, Nml = Normal

^a^IgA anti-endomysial antibody

^b^anti-insulin, IA-2, and GAD antibodies

^c^anti-GAD65 antibodies (negative for Islet Cell Antibodies)

### Response to treatment

#### Patient 1

**Fig. 4:**
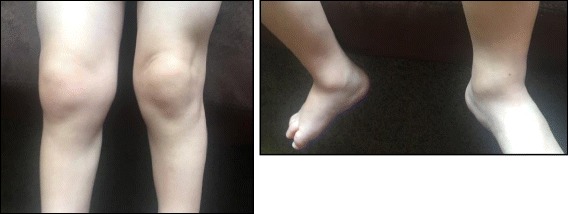
Patient 1 Arthritis. Age 3, while on adalimumab

Patient 1 was started on prednisolone (1 mg/kg/day) and naproxen (10 mg/kg twice daily) following the diagnosis of autoimmune hepatitis and arthritis. He improved but was unable to taper corticosteroids without worsening AST/ALT and arthritis. Methotrexate was avoided due to his autoimmune hepatitis. Azathioprine was added (2.5 mg/kg every other day) with improvement of AST and ALT but his arthritis persisted. Cyclosporine (6 mg/kg/day) was added as a second-line agent for autoimmune hepatitis. His AST and ALT normalized; however, his arthritis persisted. Intra-articular steroid injections of multiple joints were performed, to which he showed initial improvement; however, his arthritis flared when oral steroids were weaned. Cyclosporine was replaced with etanercept (1 mg/kg/week). Liver transaminases remained normal and his hyperpigmentation and hirsutism resolved; still, his arthritis was only partially responsive. ESR and CRP remained slightly elevated at 26 mm/h and 2.2 mg/dL. Thus, he again underwent injections of multiple joints with corticosteroids. In addition, adalimumab (10 mg every 2 weeks, weight 12.8 kg) replaced etanercept. His arthritis only improved slightly (Fig. 4), so the dose was increased to 20 mg every 2 weeks. His arthritis did not improve and his ESR and CRP worsened, so he switched to tocilizumab infusions. He now receives infusions every 2 weeks (160 mg, around 12 mg/kg) with only partial clinical response, although his ESR and CRP have normalized. He remains on prednisolone 0.35 mg/kg/day.

#### Patient 2

Patient 2 started growth hormone supplementation (83 mcg/kg/day) at 5.5 years of age and continued throughout adolescence. He was treated with weekly subcutaneous methotrexate (12.5 mg) at 6.5 years old without a clinical response during the first 4 months. Thus, cyclosporine (3 mg/kg/day) was added and soon increased to 5 mg/kg/day at 7 years old. Both medications were stopped 3 months later due to lack of clinical and laboratory improvement.

At age 8, he received prednisone (1 mg/kg twice daily) for 2 weeks with a prolonged taper as a therapeutic trial for worsening hearing. ESR reduced from 97 mm/h to 7 mm/h and his anemia resolved. His hearing continued to worsen on prednisone, which was stopped in order for him to receive cochlear implants. Prednisone (1 mg/kg/day) and cyclosporine 50 mg (4 mg/kg/day) were restarted in twice daily doses at 8.5 years old with notable improvement in thigh induration, scrotal fullness, and energy after 6 weeks. ESR again decreased and prednisone was tapered over 2 months to 0.2 mg/kg/day. He had recurring symptoms of abdominal pain and diarrhea thought to be due to partial lactase deficiency (demonstrated on biopsy of his duodenum). His height increased from below the 3rd percentile to the 3rd percentile by age 9. Cyclosporine was stopped after 1 year of treatment. His ESR remained improved on this low dose of prednisone but still elevated to 50 mm/h. Prednisone was discontinued at 13 years of age. Subsequently, his height fell to less than the 3rd percentile by age 15 years even while continuing GH.

At 16 years of age, he started adalimumab (40 mg every 2 weeks) for arthritis; however, this was stopped after 2 months due to continued symptoms and no effect on his elevated inflammatory markers. Tocilizumab (10 mg/kg IV every 2 weeks) was then started with rapid improvement in symptoms and inflammatory markers. After 2 months, his ESR improved from 66 mm/h to 11 mm/h, his CRP improved from 8.4 mg/dL to less than 0.5 mg/dL, his microcytic anemia and thrombocytosis resolved, and his thickened skin normalized. Fasting HDL improved to 14 mg/dL from 4 mg/dL pre-treatment. His height improved to the 8th percentile by age 18 years, at which time growth hormone was stopped. Despite these improvements, he had persistent hirsutism on his shins, fullness of his scrotum and mons pubis region, dyslipidemia, and IgG2 and IgG4 subclass deficiency. He was transitioned to twice weekly subcutaneous tocilizumab (162 mg twice a week, 10.8 mg/kg every 2 weeks) with continued clinical remission and normal inflammatory indices.

#### Patient 3

Patient 3 uses insulin for IDDM and follows a gluten free diet for Celiac disease. Her height remains below the third percentile for age and is paralleling the growth curve. Her diabetes has been poorly controlled with A1C levels generally above 9%, however she has not required hospitalization for diabetic ketoacidosis.

#### Patient 4

Patient 4 was treated with oral methotrexate (15 mg weekly) by her dermatologist for presumed morphea with subsequent improvement. Skin biopsy during remission did not have evidence of morphea or fibrosis. She remained off treatment for 2 years. Recently, she developed 3 new 2–3 cm hyperpigmented skin lesions consistent with previous lesions without texture change or hypertrichosis. Methotrexate was resumed at her previous dose and then switched to subcutaneous Methotrexate (25 mg weekly). Currently, her skin remains soft with no further progression. She continues to have stiff joints and an elevated ESR of 52 mm/h but no contractures or active arthritis.

#### Patient 5

Patient 5 started 25 mg of subcutaneous Methotrexate weekly at age 15 and noted shrinkage of her nasal mass and improved arthritis after 6 months. Repeat CT imaging of her nasal mass revealed mucosal thickening and a neural-based soft tissue mass within the right maxillary antrum and lateral wall of the right nasal cavity, extending through the nasal cavity into the right premaxillary subcutaneous soft tissue consistent with possible manifestations of RDD. Most recently, tocilizumab (162 mg every other week by self injector pen) was added to oral Methotrexate (25 mg weekly) with notable improvement of her skin lesions on physical exam and CT imaging, including fading back to skin tone and shrinking in size. Her ESR has decreased to 25 mm/h.

## Discussion and conclusions

Despite typical clinical findings, all five patients described here were diagnosed with H Syndrome by whole exome sequencing. Presumably, this is due to the relatively recent description of the syndrome and the small number of patients previously identified in the United States. Additionally, patients with identical mutations in *SLC29A3* frequently do not have identical phenotypes even when they are siblings, perhaps reducing the clinician’s suspicion for a Mendelian disorder. Patients 2 and 3 demonstrate this aspect of H syndrome, with the proband exhibiting many more characteristics than his sibling.

As shown in Table 1, our patients exhibit many of the manifestations as those reported in the literature, including skin findings, flexion contractures, short stature, hearing loss, and IDDM. When biopsied, our patients’ skin lesions showed dermal and deep subcutaneous inflammation with mostly uninvolved epidermis, as previously described with H Syndrome. The dermatopathologic findings from patient 2 showed findings consistent with previous reports from patients with H syndrome, including mild epidermal hyperplasia with basal pigmentation, albeit relatively mild in this case, rare dermal hemosiderin deposits, and a deep infiltrate including increased plasma cells with perivascular cuffing and histiocytes. While dermal plasma cells in RDD may express IgG4, only weak blush staining was observed in the plasma cells in patient 2, perhaps because of his IgG4 deficiency [3]. Also, the histiocytic infiltrate in RDD and H syndrome may include larger S-100 positive cells that display emperipolesis, but the histiocytes in this case were not markedly enlarged, did not contain abundant phagocytosed cells, and did not express S-100 protein [1–3, 13].

As might be expected in a report submitted by Pediatric Rheumatologists, there is an increased incidence of arthritis in our patients when compared to the literature. Unfortunately, little is known regarding the histopathology or imaging of the arthritis in this disorder. The sole MRI in this series (Patient 5) did not demonstrate synovitis, even after administration of gadolinium, while ultrasound of Patient 1 demonstrated joint effusions but no synovial thickening.

Some of the manifestations found in our patients have been rarely reported, if ever, in patients with *SLC29A3* mutations. Cardiovascular abnormalities have been described to be a relatively common finding in this disorder, but a bicuspid valve (Patient 2) has not been reported previously. Bicuspid valves, however, may occur in up to 2% of the general population and thus may not be associated with this patient’s *SLC29A3* mutation [14]. On the other hand, an absent inferior vena cava has been described once before in a patient with H syndrome [15]. The incidence of absent inferior vena cava is not well defined, however absence or stenosis of the IVC has been estimated to occur in 0.15% to 0.6% of the population and thus this rarer condition could be a manifestation of H syndrome [16]. Pericarditis (Patient 2) is also a known complication of patients with *SLC29A3* mutations [2, 4].

We observed a number of immunologic abnormalities and immune-mediated manifestations in our patients. Hypergammaglobulinemia is frequently described in patients with H syndrome, presumably as a manifestation of systemic inflammation, but selective subclass deficiency of IgG2 and IgG4 (Patient 2) is a novel finding and may or may not be a characteristic of H Syndrome. One other case report documented a low number of B memory cells, as in Patient 2, but the clinical significance of this finding is not clear [17].

Generally speaking, autoantibody formation does not seem to be characteristic of H syndrome, although one patient in the literature was originally diagnosed to have systemic lupus erythematosus on the basis of positive anti-double-stranded DNA antibodies [17]. IDDM is clearly associated with H syndrome, although in contrast to Patient 3, most of the patients have not had autoantibodies detected [18]. Serum anti-endomysial antibody is a relatively specific test for celiac disease and its presence has not been described before in patients with *SLC29A3* mutations. The occurrence of autoantibodies associated with IDDM and celiac disease in Patient 3 may reflect the patient’s genetic predisposition to developing these disorders separate from her *SLC29A3* mutations, since IDDM and celiac disease share a common genetic predisposition [19]. While hepatomegaly has been previously described in H syndrome, autoimmune hepatitis with or without autoantibodies (Patient 1) has not been described in H syndrome, but rather lymphocytic infiltration of the liver portal spaces [1]. Arthralgias and polyarticular arthritis (Patient 1 and 2) have also been described previously. It is not clear if the contracture deformities of fingers and toes found in many patients are a manifestation of synovitis or another pathologic process [2, 18].

Short stature is a very frequent finding in H syndrome. Previous reports have implicated decreased growth hormone production following stimulation and low IGF-1 levels [1]. Inflammation in Systemic Juvenile Idiopathic Arthritis is also associated with short stature and low IgF-1 levels; successful control of the inflammation with tocilizumab in Systemic Juvenile Idiopathic Arthritis is followed by an increase in growth velocity and IGF-1 [20]. Such an increase in growth velocity was seen in Patient 2 after initiation of tocilizumab therapy, although he was also receiving growth hormone treatment at the time. The cause of short stature in H syndrome may be due in part to the effect of systemic inflammation on the growth hormone/IGF-1 axis.

Given the complexity of H Syndrome, there is a lack of consensus regarding its treatment. Despite recognition of the inflammatory nature of H syndrome, treatment has been largely unsuccessful [2, 17]. Per previous reports, patients tend to respond poorly to agents directed against IL-1 or TNF-alpha such as anakinra, canakinumab and adalimumab, and colchicine or NSAIDs provide only partial relief [2, 4]. Our experience, and that of others, indicates that systemic corticosteroids have a beneficial effect on the underlying inflammation, but they are not a good long-term treatment, particularly since inflammation returns as the dose is reduced [21]. Our experience suggests that there may also be a role for methotrexate (Patients 1, 4 and 5), either alone or in combination with other medications. In this report, Patient 2 and 5 had a striking response to treatment with tocilizumab, a humanized monoclonal antibody directed against the IL-6 receptor, including reduction in hyperpigmentation and subcutaneous fibrosis, acceleration of growth velocity, and improvement in inflammatory indices, scrotal induration, and microcytic anemia. In contrast, Mistry et al. recently reported a patient with *SLC29A3* mutations who did not have clinical improvement with tocilizumab, despite normalization of the patient’s CRP [17].

With further research into the pathophysiology of H syndrome, more directed therapy will hopefully become available. There is some thought that macrophage dysfunction in patients with H Syndrome leads to a compromised immune response and elevated systemic inflammation. Hsu et al. found macrophage-dominated histiocytosis and lysosomal disturbances within macrophages present in an ENT3 null mouse model [22].

Genetic research is also expanding what we know about H Syndrome. By 2013, 21 different disease-causing mutations in SLC29A3 had been described [2]. The c.1087 > T (p.R363W) mutation in patient 1 has been described twice before in patients of Hispanic background, both with H syndrome. Similar to patient 1, both of these patients are homozygous for this mutation [23, 24]. The c.347 T > G (p.M116R) mutation in patients 1 and 2 was reported once before in a Caucasian American patient with Pigmented Hypertrophic Dermatosis with IDDM [25]. This patient was homozygous for the mutation and had parents who were fourth cousins. The other mutation in patients 2 and 3, c.610 + 1G > C, encodes a probable pathogenic splice site mutation and has not been reported before. Patients 4 and 5 are heterozygous for the c.1309G > A (p.G437R) mutation, one of the most common mutations found in patients of Arab descent [26]. The other mutation in patients 4 and 5 results in a splice site mutation at c.300 due to a G > C substitution, which has been recently described in a Moroccan patient [27]. Of interest, a different mutation at this same site (c.300 G > A) has been reported in patients of Pakistani descent with Faisalbad histiocytosis [28].

H syndrome, which seems to be more common among persons of Arab descent, has been described worldwide and should be included in the differential diagnosis of patients with short stature and systemic inflammation, particularly when accompanied by the characteristic cutaneous findings [2]. This disorder should be included among autoinflammatory syndromes, although autoimmune manifestations sometimes occur. Treatment of systemic inflammation with IL-6 blockade appears to be a promising therapeutic option in patients with H syndrome as it reduces systemic inflammation, but may not control all of the clinical manifestations. Additional treatments to consider are combination therapy with biologics and other immune suppressants. Better identification and understanding of the pathophysiology of this rare disorder may help devise earlier diagnosis and better treatment strategies.

## Abbreviations

IDDM: Insulin-dependent diabetes mellitus
RDD: Rosai-Dorfman disease
